# Detection and characterization of physiological network interactions in pulsatile motion of cranial blood vessels using real-time MRI

**DOI:** 10.3389/fnetp.2026.1701638

**Published:** 2026-02-16

**Authors:** Thorge von der Ohe, Vitali Telezki, Sabine Hofer, Peter Dechent, Martin Uecker, Mathias Bähr, Stefan Luther, Ulrich Parlitz

**Affiliations:** 1 Max Planck Institute for Dynamics and Self-Organization, Göttingen, Germany; 2 Institute for the Dynamics of Complex Systems, University of Göttingen, Göttingen, Germany; 3 Department of Interventional and Diagnostic Radiology, University Medical Center Göttingen, Göttingen, Germany; 4 Department of Neurology, University Medical Center Göttingen, Göttingen, Germany; 5 Cluster of Excellence Multiscale Bioimaging: from Molecular Machines to Networks of Excitable Cells (MBExC 2067), University of Göttingen, Göttingen, Germany; 6 German Center for Cardiovascular Research (DZHK), Partner Site Lower Saxony, Göttingen, Germany; 7 Department of Cognitive Neurology, University Medical Center Göttingen, Göttingen, Germany; 8 Institute of Biomedical Imaging, TU Graz, Graz, Austria; 9 Institute of Pharmacology and Toxicology, University Medical Center Göttingen, Göttingen, Germany

**Keywords:** arterial wall motion, CSF flow, heart and brain interactions, network physiology, real-time MRI

## Abstract

We present a robust method to assess pulsatile motion of larger cranial blood vessels in the human brain from high spatiotemporal-resolution real-time magnetic resonance (MR) imaging data. Together with percentile-based thresholding in combination with a border-detection algorithm and other empirical selection criteria, we are able to extract area time series from the pulsatile motion of blood vessels. In a proof of concept, we apply our method to the left and right vertebral arteries in a cohort of healthy subjects and extract heart and breathing rates from their pulsatile motion. Comparison to mean physiological reference values measured simultaneously with a photoplethysmogram and a breathing belt shows no differences within the scope of the measurement accuracy. Intra-subject differences for breathing rates detected in the left and right vertebral artery are high but not significant. Our findings suggest that the proposed method is suitable for assessing arterial pulsations in larger cranial vessels driven by heart or breathing rates, as part of the complex physiological network of heart–brain interactions.

## 1 Introduction

Arterial pulsations (Iliff et al., 2013; Mestre et al., 2018; Hablitz and Nedergaard, 2021) and respiration (Dreha-Kulaczewski et al., 2015) are assumed to drive bulk flow of cerebrospinal fluid (CSF). This flow is believed to play a crucial role in the brain’s waste disposal process (Nedergaard, 2013; Rasmussen et al., 2022; Mestre et al., 2020). CSF flow drives the clearance of tau, amyloid- β , α -synuclein, and other metabolites, the accumulation of which is associated with diseases such as Alzheimer’s disease and Parkinson’s disease (Iliff et al., 2012; Patel et al., 2019; Lundgaard et al., 2017; Stefanis, 2012).

It has been demonstrated that alteration in arterial wall motion due to acute arterial hypertension dramatically reduces CSF flow speeds and increases CSF backflow (Mestre et al., 2018). It is speculated that such altered CSF flow also reduces parenchymal waste transport and, therefore, induces a risk factor for cognitive diseases, such as Alzheimer’s disease (Benveniste, 2018; Rasmussen et al., 2022). In addition, heart, brain, and blood vessels comprise a complex network of physiological interactions (Ivanov, 2021). Analysis of vessel pulsatility and the potential impact of heart and breathing rates on it can provide further insights into the dynamic bidirectional coupling between the heart and the brain (Bartsch et al., 2015; Lin et al., 2016).

So far, because of its higher spatial and temporal resolution and reduced cost compared to magnetic resonance imaging (MRI)-based methods, ultrasound-based imaging techniques such as neurovascular ultrasound (nvUS) and transcranial Doppler ultrasound (TDU) are clinically established methods for assessing cranial vessels noninvasively (Markus, 1999; Wagshul et al., 2011). However, these techniques are often limited to measuring single selected vessels at a time and are subjected to variability of the operator’s expertise. In addition, insonation of cerebral arteries of interest is not possible in 10%–20% of subjects because of suboptimal insonation angles for TDU (Baumgartner, 2006). Furthermore, traditional nvUS measurements assume circular cross sections of the measured vessels (Maier et al., 2018). Moreover, nvUS performance can be limited by location (e.g., distal extra- and intracranial part of internal carotid artery) and other anatomical variations among subjects (e.g., short neck or obesity), and it requires a hyper-extended head during examination (Maier et al., 2018), which reduces subject comfort.

Although time-resolved phase contrast MRI (4D PC-MRI) has been used to quantify vascular streaming and pulsation (van Hespen et al., 2022; Xie et al., 2024), this method relies on combining information from many heart cycles and, therefore, assumes periodicity of the heartbeat. In addition, to account for the highest expected flow velocities, some 4D PC-MRI acquisition parameters are required to be specified before image acquisition. A wrong choice may lead to loss in image quality or introduce velocity aliasing artefacts. Despite current efforts to reduce scan times, 4D PC-MRI is time-consuming (Gottwald et al., 2020; Peper et al., 2020).

In this study, we present an alternative strategy to robustly characterize the pulsatile motion of larger cranial vessels using real-time MRI (Uecker et al., 2010). In a proof of concept, we show how the extracted pulsation rates of the left and right vertebral arteries (VAs) compare with other physiological recordings of heart and respiratory rates in a healthy control group.

## 2 Materials and methods

This study was approved by the local Ethics Committee of the University Medical Center Göttingen (no. 37/3/19). Written informed consent was obtained from each participant.

### 2.1 Study design

**FIGURE 1:**
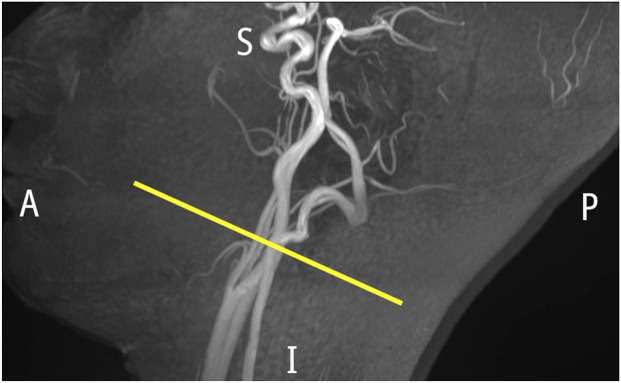
Maximum intensity projection of the arterial vessels (A, anterior; P, posterior; S, superior; I, inferior). Slice position for real-time MRI measurement is indicated by the yellow line.

This study consists of a group of healthy subjects ( n=20 , 9 male, 26±6 year). Each participant underwent one session of MRI performed at 3T (Prisma Fit, Siemens Healthineers, Erlangen, Germany), equipped with a 64-channel head coil. The plane of imaging was placed orthogonal to the internal carotid artery (ICA) by an experienced operator, directly above its bifurcation point (see Figure 1). Real-time radial fast low-angle shot (FLASH) MRI ( TR=3.08 ms , TE=2.04 ms , flip angle=10° ) (Uecker et al., 2010) was conducted under free breathing without physiological gating. We acquired high spatial ( 0.8×0.8×6 mm^3^) and temporal (25 images per second) resolution real-time MRI data for 60 s, resulting in 1,500 images. In parallel, we recorded heart and breathing rates of subjects using a finger photoplethysmograph (PPG) (Siemens, 200 Hz) and a breathing belt (Siemens, 50 Hz), respectively.

To demonstrate modulation of arterial pulsatility through respiratory activity, we performed real-time MRI for two additional subjects (both male, 19 years and 23 years). Breathing instructions were communicated via a visual paradigm with specified symmetric inhalation and exhalation phases. We investigated three paradigms—(1) slow-breathing paradigm, with 12 breathing cycles per minute, (2) fast-breathing paradigm, with 24 breathing cycles per minute, and (3) breath-hold paradigm, aiming for 30 s. For each paradigm, we recorded MRI and physiological signals over 90 s. For the slow- and fast-breathing paradigms, only the central 60 s of data were analyzed, allowing the subjects’ heart rates to stabilize after the breathing transition. For the breath-hold paradigm, analysis was confined to the actual breath-hold interval.

### 2.2 Analysis pipeline

Study data are stored and managed using our research data management tool (Telezki et al., 2024). To extract pulsatory motion of blood vessels from our recorded real-time MR images, we use the following steps.

**FIGURE 2:**
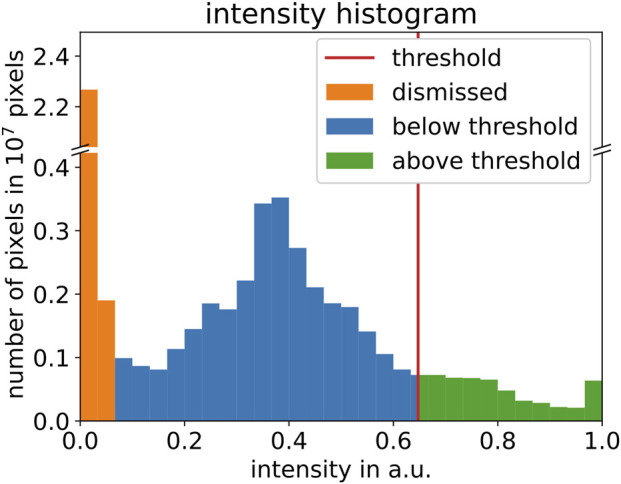
Normalized intensity histogram of one real-time MRI dataset, illustrating threshold calculation. Orange bins (intensity values below 1/15 ) highlight the dismissed background. The threshold based on the 85th percentile of the remaining foreground is marked with a red vertical line. Blue (green) bins highlight intensity values below (above) the threshold.

First, the real-time MRI dataset from each session is normalized to values between 0 and 1 through linear rescaling. Second, we apply an empirical intensity threshold to binarize each image. As illustrated in Figure 2, selection of the intensity threshold is based on the intensity histogram for each normalized dataset. Here, we assume a uniform distribution of intensity values within each bin. Pixels with intensity values below 1/15 , corresponding to the first two bins (orange), are classified as background of the dataset and are dismissed. The threshold is chosen as the 85th percentile value of the foreground. We achieved the best qualitative results with an 85th percentile threshold. However, any threshold between the 80th and 90th percentiles yielded comparable results for the dataset analyzed here. Pixels with intensity values above the threshold (green) represent blood vessels and some artifacts.

**FIGURE 3:**
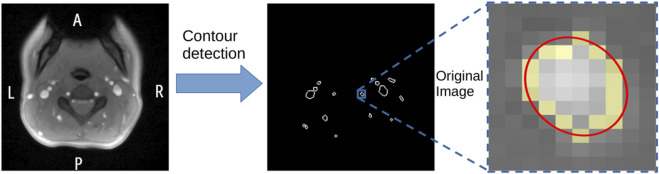
Illustration of blood vessel detection: left: single exemplary frame of MRI data in axial orientation (A, anterior; P, posterior; L, left; R, right); center: detected contours for the same frame (see step 2.2); right: extracted original image around a single detected blood vessel. The yellow pixels depict the detected contour. The red line shows ellipse fitted to detected contour.

Third, to further distinguish blood vessels from artifacts, we use a contour-detection algorithm (*openCV*, Bradski, 2000) on each binarized frame of the MRI dataset. To identify contours representing blood vessels, we fit an ellipse to each detected contour, resulting in the major and minor axes and the area of the ellipse. These parameters are then used to filter the detected contours based on the following empirical criteria. The area of the ellipse has to lie within 5 and 500 times the area of a single pixel (3.2 mm^2^–320 mm^2^). The quotient of the lengths of the principle axes has to lie within 2/7 and 7/2 . Other contours are too small, too big, or do not have the expected eccentricity to be considered a blood vessel and are therefore omitted from further analysis. The steps for detecting blood vessels in our real-time MR images are summarized in Figure 3. Videos illustrating these individual steps can be found in the Supplemental Material.

**FIGURE 4:**
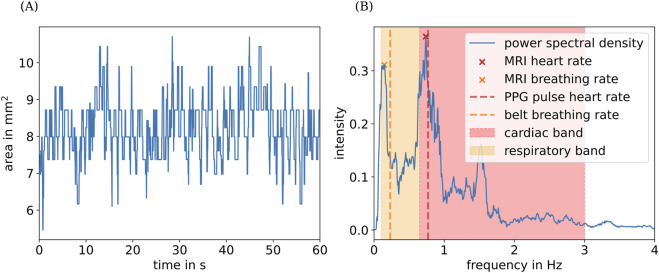
**(A)** Extracted cross-sectional area for the right vertebral artery of one subject over time. **(B)** Its calculated power spectral density, along with the mean heart and breathing rates extracted from physiological data (red and orange vertical lines) (see Section 2.2 and Section 2.3). Orange and red highlighted areas indicate the physiological ranges of breathing and pulse rates used for peak detection. The peak in the spectrum at approximately 1.5 Hz corresponds to the first harmonic of the cardiac component of the MRI signal.

In addition, we assess each remaining contour for overlaps over five consecutive frames. Contours that overlap by at least 50% are assumed to describe the same blood vessel and are therefore grouped together, resulting in a time series for each contour. Finally, we approximate the area of the detected blood vessel contour within each time series by the area of its fitted ellipse, resulting in an area time series for each detected blood vessel. An example of the resulting area time series is shown in Figure 4A.

We then proceed to determine the dominant frequencies of the area time series as follows. First, we apply a band-pass filter (0.1 Hz–4 Hz). Second, we compute power spectra of the filtered area time series using a multi-taper power spectral estimation method (Thomson, 1982), implemented in the R package spec.mtm (Rahim et al., 2014). Finally, we determine the maximum frequencies within the respective ranges of breathing (0.1 Hz–0.65 Hz) and heart rates (0.65 Hz–3 Hz) , motivated by physiological values for resting adults (Chourpiliadis and Bhardwaj, 2022; Nanchen, 2018), as illustrated in Figure 4B.

### 2.3 Physiological data

We analyze the recorded PPG heart rate and breathing belt data (see Section 2.1) using the ppg_process and rsp_process functions (*NeuroKit2*, Makowski et al., 2021), as follows. PPG heart rate data are preprocessed using a third-order bandpass Butterworth filter (0.05 Hz–8 Hz) (Butterworth, 1930), followed by a peak detection algorithm (Elgendi et al., 2013). The detected peaks are used to calculate pulse rates based on the pulse period.

Breathing belt data are preprocessed using a second-order bandpass Butterworth filter (0.05 Hz–3 Hz) . Afterward, peaks are detected using a zero-crossing algorithm with an amplitude threshold (Khodadad et al., 2018). Physiological consistency is realized by enforcing that a trough lies between two peaks and a peak between two troughs. The resulting detected peaks are used to calculate breathing rates based on the breathing period (Makowski et al., 2021).

We then calculate the mean physiological pulse and breathing rates from our physiological recordings as reference values.

## 3 Results

In a first proof of concept, we limit our analysis to the left and right VAs. This artery is an appropriate choice because of its typically isolated position from other blood vessels such as the jugular vein or the internal or external carotid arteries. In addition, the typically smaller diameter of the VA compared to other cranial arteries provides a rough assessment of size and resolution limitations.

### 3.1 Controlled breathing

To demonstrate modulation of arterial pulsatility through respiratory activity, we first evaluate the acquired data from our controlled breathing sessions. Here, we applied the three paradigms, namely, slow-breathing, fast-breathing, and breath-hold paradigms. Additionally, we compare these results to their respective baseline measurements under free breathing.

**FIGURE 5:**
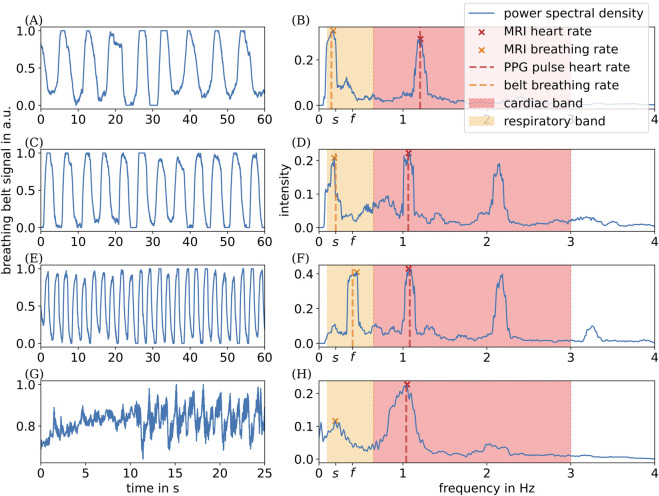
Respiration–time curves measured with the breathing belt for different paradigms: **(A)** free-breathing; **(C)** slow-breathing (12 cycles/min); **(E)** fast-breathing (24 cycles/min); **(G)** breath-holding, and the corresponding spectral analyses of the pulsatile motion of the left VA **(B,D,F,H)**. s represents the frequency corresponding to 12  cycles/min, and f represents the frequency corresponding to 24  cycles/min.

To assess whether the subjects complied with the breathing instructions, we analyze the recorded breathing belt data, as presented in Section 2.3. As shown in Figures 5C,E, the prescribed breathing rates of 12 (24) respiratory cycles per minute were achieved with minimal deviation. Our subjects were able to hold their breath for up to 25 s.

Using the analysis steps presented in Section 2.2, we extract heart and breathing rates from the pulsatile motion of the left and right VAs visible in our real-time MRI data under varying breathing conditions for one of the two subjects. We dismissed evaluation of real-time MRI data for one subject because of the subject’s atypical arterial anatomy. Here, the VA was positioned unusually close to adjacent vasculature, precluding reliable segmentation. The extracted area–time curves and their corresponding power spectral density for one subject are shown in Figure 5.

Under free-breathing conditions (Figures 5A,B), we extracted two dominant frequencies from the area–time signal: one within the respiratory band (orange), reflecting respiratory modulation, and another within the cardiac band (red), reflecting cardiac modulation. Both dominant frequencies align closely with the simultaneously recorded physiological signals (vertical dashed lines). Under slow- and fast-breathing conditions (Figures 5C–F), the dominant respiratory frequencies extracted from the area–time signal match the prescribed breathing rates very closely, as indicated by the respective tick labels. These findings clearly demonstrate a causal modulation through both respiratory and cardiac activities of the pulsatile motion of the left and right VAs.

However, during breath-hold conditions (Figures 5G,H), we noticed that contributions to the power spectral density below the physiological respiratory band are comparable in magnitude to those within the respiratory band. This pattern seems to be unique for the breath-hold experiment. When non-physiological components outside the respiratory band contribute in the same way as genuine physiological contributions within the respiratory band, our method cannot clearly distinguish between true physiological modulation and other effects.

### 3.2 Free breathing

Next, we extract heart and breathing rates from the pulsatile motion of the left and right VAs visible in our real-time MRI data and compare those to the simultaneously recorded physiological values as reference under free-breathing conditions (n=20) . We omitted one MRI dataset due to its low contrast. With our proposed method, we were able to extract area time series data from 36 out of the remaining 38 vertebral arteries, thereby resulting in at least one correctly detected VA per subject.

**FIGURE 6:**
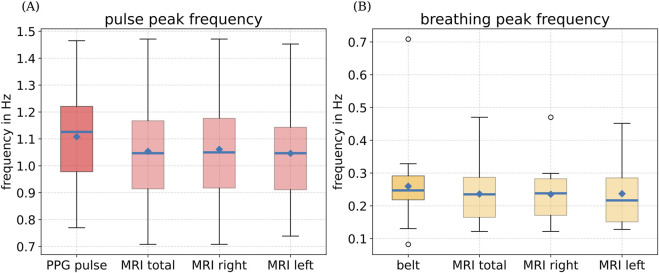
Boxplot for **(A)** heart rates and **(B)** breathing rates from physiological recordings using PPG and a breathing belt and as extracted from pulsatile motion of left and right VAs from real-time MRI. Blue lines denote the median, and the blue dots represent the mean. Boundaries indicate the 25th and 75th percentiles. Whiskers show minimum and maximum values, whereas outliers are depicted with circles.

As shown in Figure 6, pulse and breathing rates estimated with external sensors (PPG or breathing belt) are similar to rates estimated from pulsatile motion of left and right VAs from real-time MRI following our proposed method. In fact, we report the same mean heart rates (PPG: 1.11±0.16 Hz; MRI: 1.07±0.19 Hz) and breathing rates (belt: 0.25±0.05 Hz; MRI: 0.26±0.10 Hz) averaged over all subjects, within the scope of the measurement accuracy.

**FIGURE 7:**
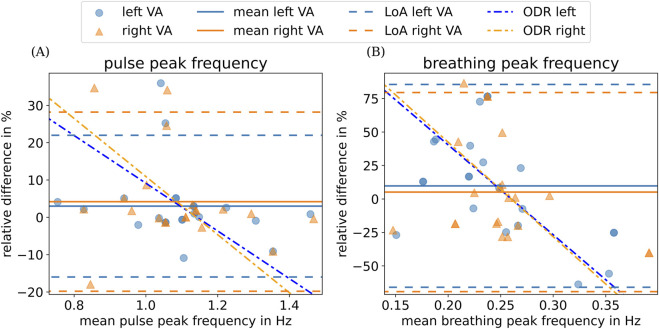
Bland–Altman plot depicting **(A)** the relative difference between the mean frequency measured using PPG and the peak heart frequency obtained from area time series and **(B)** the difference between the mean frequency measured using a breathing belt and the peak breathing frequency obtained from area time series for left and right vertebral arteries (VAs). Corresponding mean differences over all subjects are indicated by colored horizontal lines. The limits of agreement (LoAs), which correspond to the mean ±1.96 times the standard deviation, are indicated by the dashed colored lines. The dashed dotted lines show the result of an orthogonal distance regression (ODR) using an affine linear function for each side.

To further evaluate the extent of agreement between heart and breathing rates extracted from real-time MRI data with their physiological reference values measured with PPG and breathing belt, we present their respective absolute relative differences in a Bland–Altman plot (Figure 7).

For heart rate data (Figure 7A), mean relative differences are low for both left (3 %) and right (3.7 %) VAs, indicating only a small bias. In addition, heart rates extracted from the pulsatile motion are similar between left and right VAs for each subject (low intra-subject variability), indicating that we can robustly detect the mean heart rate from our real-time MRI data. This finding is supported by a Pearson correlation value of 0.78 between the mean PPG pulse frequency and the MRI pulse frequency.

For breathing rate data (Figure 7B), although mean relative differences are low for the right VA (−1.9 %) , mean relative differences for the left VA indicate a moderate bias (9.8 %) . Here, the detected intra-subject differences between left and right VAs are high but not significant (paired t-test, $\left.p=0.56\right)$ . High relative differences can be partially attributed to the short acquisition time of 60 s. During this period, we observe on average 15 breathing events according to breathing belt data, leading to larger relative errors when calculating the mean breathing rate. Physiological intra-subject differences between left and right VAs (Mitchell and McKay, 1995; Spasojević et al., 2020) or body position during MRI acquisition can partially account for the observed intra-subject variability. The Pearson correlation between the mean breathing belt frequency and the MRI breathing frequency is 0.14.

To quantify the present proportionality bias, we applied an orthogonal distance regression (ODR) using a linear function. For the heart rate, this results in a slope of −64±23 % Hz^−1^ for the left vertebral artery and −78±28 % Hz^−1^ for the right artery. For the breathing rate, this results in a slope of −672±168 % Hz^−1^ for the left vertebral artery and −706±227 % Hz^−1^ for the right artery. These resulting slopes for both heart rate and breathing rate data indicate a pronounced negative bias (dashed–dotted lines); however, the confidence intervals are relatively wide. Additional experiments are required to determine the statistical significance of this effect.

## 4 Discussion and conclusion

Our demonstrated approach allows us to automatically detect and extract blood vessels from real-time MR images. The proposed method is sufficiently robust to produce area time series for individual blood vessels, which can be used in further analysis steps. As a proof of concept, we aim to extract heart and breathing rates from the pulsatile motion of the left and right VAs.

A comparison of mean heart rates from physiological recordings with those extracted from pulsatile motion of the left and right VAs recorded by real-time MRI demonstrated very good agreement across the cohort (see Figure 6A). In addition, the strong Pearson correlation of 0.78 indicates that the proposed method can reliably estimate heart rates directly from 60 s of real-time MRI data without the need for external sensors. However, we note that the limits of agreement are substantially broader than the standard regulatory thresholds for consumer-grade PPG sensors (ANSI, 2016).

Cohort-averaged breathing rates extracted from pulsatile motion are in good agreement with their physiological reference values recorded using a breathing belt, whereby the limits of agreement are relatively wide, indicating a potential discrepancy between both methods, which requires further investigation.

Next to the short acquisition times, our study has other limitations potentially influencing the results. First, although estimation of resting state heart rates from photoplethysmogram recordings is known to be robust and accurate (Iyriboz et al., 1991; Elgendi et al., 2013), estimation of breathing rates using a breathing belt relies on measurements of relative changes in the abdomen volume. Potential shallow breathing, tightness and placement of the belt, overall motion, or breath hold events in some subjects affect the overall signal quality and therefore estimation of the reference breathing rates. This is reflected in a high measured relative standard deviation of approximately 20%. We recommend monitoring the respiratory signal using an integrated spine coil sensor (e.g., BioMatrix Respiratory Sensor, Siemens) in the future, leading to improved subject comfort and potentially improved signal quality (Wilding et al., 2024). In addition, longer acquisition times might aid in recording of longer free breathing events, overall improving the estimation of reference breathing rates. Second, estimation of pulsatile motion from real-time MRI data is limited by spatial and temporal resolution. The choice of our acquisition parameters allows visualizing motion of larger cranial arteries such as internal carotid, basilar, or vertebral arteries. In our analysis, we omit frequencies above 4 Hz , corresponding to a Nyquist rate of 8 Hz, which sets the minimal temporal sampling rate to avoid aliasing (Shannon, 1949). With a temporal sampling rate of 25 Hz, we are well above the Nyquist rate in this study. Reliable detection of smaller vessels would benefit from an increased spatial resolution, at the cost of temporal resolution. In general, rare occasion of swallowing events and subject-specific close proximity to other blood vessels or bones can affect reliability of our method to extract isolated pulsatile motion. Third, although the choice of a percentile-based global threshold for contour detection works reliably for our sample size, local thresholding (Niblack, 1985) or a combination of local and global thresholding algorithms might be more suitable with a larger and more diverse cohort. Finally, we focused on the vertebral artery as a proof of concept in this study. A comparison with other larger cranial arteries and a statistical evaluation of inter- and intra-subject variability in a larger cohort would be desirable. Our proposed method has the potential to extract pulsatile motion from all visible blood vessels simultaneously. This approach might represent a new method to study the complex network of (intra-cranial) blood vessels, in relation to physiological recordings such as heart and breathing rates, blood pressure, and cardiac electric activity.

## Data Availability

The datasets presented in this study can be found in online repositories. The names of the repository/repositories and accession number(s) can be found below: the source code is available at https://codeberg.org/tvdo/pulsating_blood_vessels, and one example dataset supporting the conclusions of this article is available at https://doi.org/10.25625/JIHTIJ.
