# Supplementary material for: Detection and characterization of physiological network interactions in pulsatile motion of cranial blood vessels using real-time MRI
Source: Front Netw Physiol. 2026 Feb 16;6:1701638. doi: 10.3389/fnetp.2026.1701638 (PMC12950750; doi:10.3389/fnetp.2026.1701638)
Supplement: Supplementary file 2 [file DataSheet1.pdf]

Supplementary Material

1 CONTENT OF SUPPLEMENTAL VIDEOS

The supplemental videos illustrate the result of the sub-steps of the analysis pipeline described in Sect. 2.2. A short explanation of the content of each video can be seen in Table S1.

filename	description
movie_1.mp4	an extract of the recorded data for one subject
movie_2.mp4	same dataset as 'movie_1.mp4' after application of the calculated threshold
movie_3.mp4	boundaries of the contours detected within the dataset seen in 'movie_1.mp4'
movie_4.mp4	detected contour corresponding to the left vertebral artery as well as the original video depicting that artery
movie_5.mp4	detected contour corresponding to the right vertebral artery as well as the original video depicting that artery

Table S1. Description of processing steps illustrated by the supplemental videos.
